# Cadaver versus simulator based arthroscopic training in shoulder surgery

**DOI:** 10.3906/sag-2011-71

**Published:** 2021-06-28

**Authors:** Gazi HURİ, Mert Ruşen GÜLŞEN, Ece Belen KARMIŞ, Doğaç KARAGÜVEN

**Affiliations:** 1 Department of Orthopedics and Traumatology, Faculty of Medicine, Hacettepe University Ankara Turkey; 2 Medical Doctor, Edremit State Hospital, Balıkesir Turkey; 3 Medical Doctor, İstanbul Tuzla State Hospital, İstanbul Turkey; 4 Department of Orthopedics, Faculty of Medicine, Ufuk University, Ankara Turkey

**Keywords:** Arthroscopy, simulator, education, shoulder, coronavirus

## Abstract

**Introduction:**

There are few studies that compare the cadaver dissections with the medical simulators in means of talent improvement. Therefore, the aim of this study is to find out if using cadaver dissections is still the golden standard for surgical training or using the medical simulators in surgery could replace cadaver dissections.

**Materials and methods:**

The study is conducted during the European Orthopaedics & Traumatology Education Platform accredited Shoulder Club International Cadaver Course including a number of 34 orthopedics trainees. The participants were randomly divided into two groups to be trained with the simulator (Group 1) and on cadavers (Group 2), followed by a test performed on shoulder arthroscopy simulator (Virtamed ArthroS, Switzerland). There was no conflict of interest before, during, or after the study. Informed consent was obtained from all individual participants included in the study.

**Results:**

Group 2 had statistically significant higher simulation overview procedure time values than Group 1 (p < 0.05), the meaning of which is participants trained with the simulator completed the given tasks in a shorter period of time. Group 2 had statistically significant higher scratching of humerus cartilage values than Group 1 (p < 0.05), which means that participants trained with simulation have less scratching done on the humerus cartilage than the participants trained on a cadaver.

**Conclusion:**

To the best of our knowledge, this study is the first one to compare virtual reality (VR) simulators with cadavers for surgical education in an objective manner, while using qualitative and quantitative data. According to this study, it is possible to state that VR simulators are just as effective as cadavers in means of training subjects. As medical education will face a total change all around the world after the COVID-19 pandemic, this study has the potential to be an important guide during and after this period.

## 1. Introduction

The world has encountered a new type of Coronavirus outbreak by the end of 2019, which has evolved into a pandemic in 2020. Coronavirus is an RNA virus capable of infecting humans and several species of animals [1]. Main way of transmission is droplet spread [2] in between humans within the distance of one meter. The disease can progress without presenting any symptoms initially while causing pneumonia and viral sepsis [3] later on, especially among members of high-risk groups. It has an estimated mortality rate of 3.4% globally WHO (2020) WHO Director–General’s opening remarks at the media briefing on COVID-19 [online]. Website https://www.who.int/dg/speeches/detail/who-director-general-s-opening-remarks-at-the-media-briefing-on-covid-19---3-march-2020 [accessed at 15.05.2020]., which is even higher among individuals who have comorbid diseases. Not contaminating with the virus has become the priority of managing the disease, which can be achieved by using self-protective equipment and social distancingCDC (2020) How to Protect Yourself & Others [online]. Website https://www.cdc.gov/coronavirus/2019-ncov/prevent-getting-sick/prevention.html. [a ccessed at 15.05.2020]. . The authorities in many countries are promoting social distancing and isolation by taking decisions accordingly. Declaration of curfew and suspension of gathered educational activitiesUNESCO (2020) 290 million students out of school due to COVID-19: UNESCO releases first global numbers and mobilizes response [online]. Website https://en.unesco.org/news/290-million-students-out-school-due-covid-19-unesco-releases-first-global-numbers-and-mobilizes [accessed at 15.05.2020]. are a few to count.

This pandemic has deeply affected the field of medicine as well. Together with the medical diagnostic and therapeutic methods, medical education has also been affected in a negative way. There is increasing demand for new, accessible, sustainable and distant educational models and methods.

Many institutions have been trying to keep educational activities ongoing by providing online lectures and webinars as this is considered the only way of sustainability in current circumstances. Medical education and residency training, however, is different regarding its element of “Practice”, which is lacking currently as elective surgeries together with outpatient clinics are suspended in many hospitals due to Coronavirus outbreak. 

Practicing as much as possible is an essential part of surgical residency in medicine. This essential practice can be done on the patient him/herself. Other options, which might be considered better in many ways [4], is to gain such experience before confronting the patient with the help of the cadavers or virtual reality (VR) simulations. Having hands-on experience on the patient provides invaluable improvement to the surgeon; however, there are some downsides of this kind of experience as such higher complication rates and bigger economic burden are observed to occur when the operation is done by a less experienced surgeon [5,6] and vice versa; more experience is associated with shorter operation time and less blood loss [7]; a supervisor is required during surgeries and this might be considered as a time loss for more experienced supervisor; trainee is not able to practice every scenario on patients.

When practice done on cadavers is considered, which is accepted as golden standard [8], it offers an opportunity to gain experience without causing any risk for patients. However, trainings done on cadavers are expensive [9]; there is danger of disease transmission throughout the process [10]; the practice is not repeatable because cadaver better be disposed when training is done due to disease transmission risks and disrupted anatomy; there are not enough cadavers for every resident to do training because of economic limitations. There are also limitations considering the preservation of cadavers. Laboratories that have cadaver, require to employ specialists who are trained to look after the specimens. Cadavers that would be used for laparoscopic training purposes require to be processed with one of two techniques: Cryopreservation and Embalming [11]. Another important limitation is that, trainings on cadavers is generally done with a crowded population as they require at least one instructor, one technician and two trainees to be present. This can be considered as a limitation because after COVID-19 pandemic is, it is high likely possible that the number of highly populated educational activities will be decreased and new regulations are going to take place. Importance of this limitation is highlighted with the current pandemic. 

The method which has been evaluated in this article as an alternative to cadavers is VR simulation. Potential benefits that can be taken advantage of are as following:

- Residents are able to improve themselves anytime, anywhere without restrictions. When compared to cadaver trainings, there is no need to find/buy cadavers nor special conditions to preserve them.

- One VR simulation device could be used by several residents for many years unlike cadavers.

- There would be less need for an experienced supervisor as the software on simulation could show the trainee where she/he made the mistake and give appropriate feedback. Providing free time for experienced surgeons to perform surgery on a real patient.

- There is less to none danger of disease transmission while practicing on simulations as there is not any organic tissue needing to be taken care of.

- Training regularly with simulators would speed up the learning curve. Thus, trainees who have access to VR simulations will be ready to operate on real patients sooner [12].

- Trainees are able to train with VR simulators all alone, without needing anyone else to supervise them, which is a significant advantage in means of social distancing.

Today in most of the countries around the world, the medical education system consists of theoretical lessons, followed by clerkships where the doctors are in the clinics to observe and learn. Ratio of cadaver training in medical education is not high as in the surgical residency education majorly due to economic concerns. Majority of medical graduates do not possess knowledge about arthroscopic/laparoscopic methods as they do not get the chance to investigate the inner functional anatomy of joints or internal organs. 

If medical students and residents do practice with VR simulations during their first year, they would be more familiar with the anatomy and procedures when they start performing operations on patients. Thus, shorter operation times would be the outcome, and there will be less complications as long operation time is correlated with more complications [13].

There is a solution for sustaining practical education during these unfavorable conditions: Virtual Reality (VR) Simulations. It is achievable to make use of the time well by gaining necessary experience with VR, without disturbing social isolation. This new model of education might help us to minimize the negative effects of such conditions on medical education and residency training. After this pandemic gets under control, there will be almost a year passed without any practice opportunity. If VR simulations are used effectively, this problem can be countered with. However, the foremost question is “would VR simulations be as useful as the golden standard of surgical training, cadavers?”

VR simulations hold a great potential when compared with the cadaver training, which is accepted as the gold standard for surgery training. However, both have downs and ups. There are few studies that compare the cadaver dissections with the medical simulators in means of talent improvement. A study that is comparing these two modalities with many aspects would be much helpful. Therefore, the aim of this study is to find out if using the cadaver dissections is still the golden standard for surgical training or using the medical simulators in surgery could replace the cadaver dissections.

## 2. Materials and methods

The study is conducted during the European Orthopaedics & Traumatology Education Platform accredited Shoulder Club International Cadaver Course including a number of 34 orthopedics trainees. The participants were randomly divided into two groups to be trained with simulator and on cadavers, followed by tests performed on shoulder arthroscopy simulator (Virtamed ArthroS, Switzerland). Although divided randomly, groups were homogenized as there was no statistically significant difference spotted regarding the answers given to surveys collected from participants. Percentage distribution of answers can be reviewed from Table 1. 

**Table 1:** Information about the participants.

1. Demographics	
Average age	39
Sex	100% male
	80% Right-handed
	15% Left-handed
Dominant hands	5% Ambidextrous
	50% Turkey
Country of residency	45% Europe
	5% UAE, Dubai
2. Hobbies	
Playing sports as a child	65% (with the average starting age of 8,5)
Playing sports now	25% (Football, basketball, cycling, fitness, running, table tennis)
Playing music instrument	20% (Guitar, violin, piano)
Playing video games	35%
Like driving maneuvers	85%
3. Past of simulation training	
No VR training before	34%
Less than 2 h	63%
More than 10 h	3%
4. Number of shoulder arthroscopies per year	
More than 30 shoulder arthroscopies	40%
Between 10 and 30	35%
Less than 10	25%

### 2.1. Demographics

Demographic information is collected from each participant before the study via the survey. Questions asked in the survey can be reviewed from Table 2.

**Table 2:** Survey questions.

Demographic information
1. Date of birth
2. Gender
3. Dominant hand
4. In which country did you get your residency training ?
5. In which city did you get your residency traning ?
Personal traits
6. Did you do any sports when you were a child ?
7. If yes, what sport was it ?
8. At which age did you start doing sports ?
9. Do you still do sports; if yes, how often ?
10. Do you play video games; if yes, how often ?
11. Do you play any musical instrument; if yes, which one is it ?
12. Do you like performing driving maneuvers ?
Wellbeing
13. How many hours did you sleep yesterday ?
14. How many hours did you sleep last three days ?
15. Are you in a good physical condition ?
16. Do you have any kind of problems concerning your health that would restrict your movements or daily activites ?
17. Do you have any problems that would cause you to distract easily ?
Experience on surgery
18. How many knee arthroscopies do you perform per year ?
19. How many shoulder arthroscopies do you perform per year ?
About medical simulations
20. Did you ever get any training with surgery simulators ?
21. If yes, how many hours did you get ?
22. Do you believe if the artificial reality and simulators are beneficial for arthroscopy and surgery trainings ?

 The mean age was 39. They all have stated they were in a good physical condition which did not restrict their movements or daily activities of any kind; they all had their sleep well in the last week (average 20 h), and there was not any problem that might affect their levels of attention. Fifteen percent of the participants were left-handed, 5% were ambidextrous, and 80% were right-handed. Countries of residencies were Turkey, Europe, and Dubai. 65% of participants were playing sports when they were child and the average age of starting was 8.5. Dominant sport was football followed by basketball. Twenty-five percent (25%) of participants were still playing sports, which is most frequently once a month. Twenty percent (20%) were playing a music instrument including guitar, violin, and piano. Thirty-five percent (35%) were playing video games once a week or once a month. Eighty-five percent (85%) did like driving maneuvers such as drifting. Sixty-five percent (65%) had simulator training only once in their life, which was less than one hour. 35% never had any simulation training before and it was their first time with simulators during this study. Forty percent (40%) of the participants stated that they are doing more than 30 shoulder arthroscopies per year. Twenty-five percent (25%) stated the same number as less than 10 per year and 35% stated the number is between 10 and 30 per year.

### 2.2. Training module

**Figure 1:**
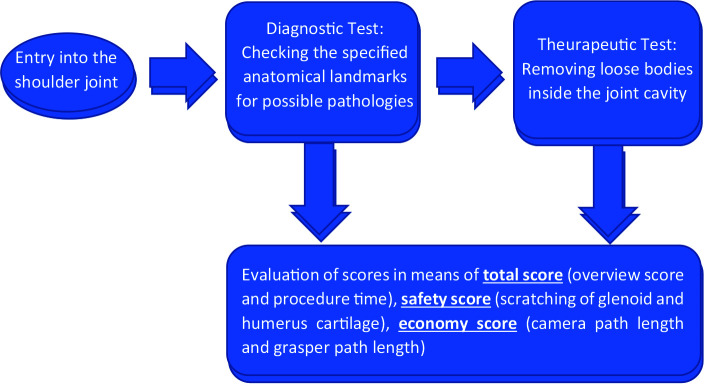
Participants are having VR anatomy education. Written informed consents were obtained for the use of photographs.

After training, both groups have undertaken the standard knowledge and skill test which we named as the “talent test” on the VR simulator (Virtamed ArthroS). The first group trained with shoulder simulator (Virtamed ArthroS) (Figure 1) before the talent test while the other group took the same talent test after being trained with cadavers. Both simulator and cadaver training ended in 20 min for each individual and the talent tests on the simulator was limited to 15 min.

Training on cadaver and simulation covered similar steps and skills as following: 

Entering the shoulder joint, visualization of designated anatomic landmarks (Glenohumeral joint, biceps tendon, supraspinatus insertion, infraspinatus, subscapularis, humeral head, glenoid cartilage, dorsal labrum, superior labrum, anterior medial labrum, inferior labrum, subacromial joint, acromion, coracoacromial joint, acromioclavicular joint), and removing loose bodies inside joint cavity.

**Figure 2:**
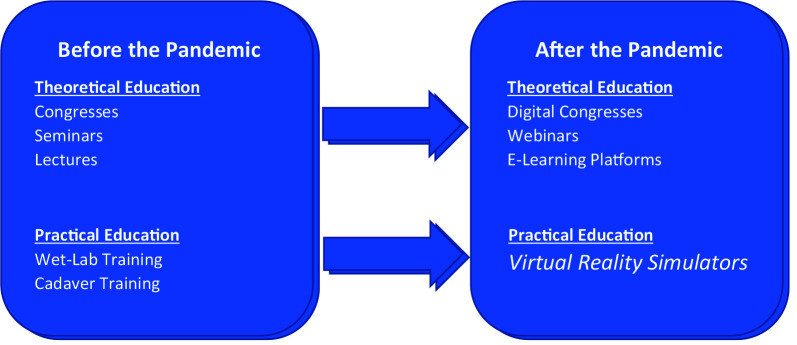
One of the participants is performing the talent test with the VR Simulator. Written informed consents were obtained for the use of photographs.

Following the training module, both groups were subjected to talent test (Figure 2). 

### 2.3. Talent Test

The talent test consists of two parts: (a) diagnostic (b) therapeutic. In the diagnostic part, the participants were asked to check the anatomical landmarks: glenohumeral joint, biceps tendon, supraspinatus insertion, infraspinatus, subscapularis, humeral head, glenoid cartilage, dorsal labrum, superior labrum, anterior medial labrum, inferior labrum, subacromial joint, acromion, coracoacromial joint, acromioclavicular joint, and mark the pathologies. The aim of this test was to figure out if the participants could visualize the entire landmarks and find any possible pathologies inside the shoulder joint. Furthermore, in the therapeutic part, the participants were asked to remove loose bodies in the shoulder within 5 min.

**Figure 3:**
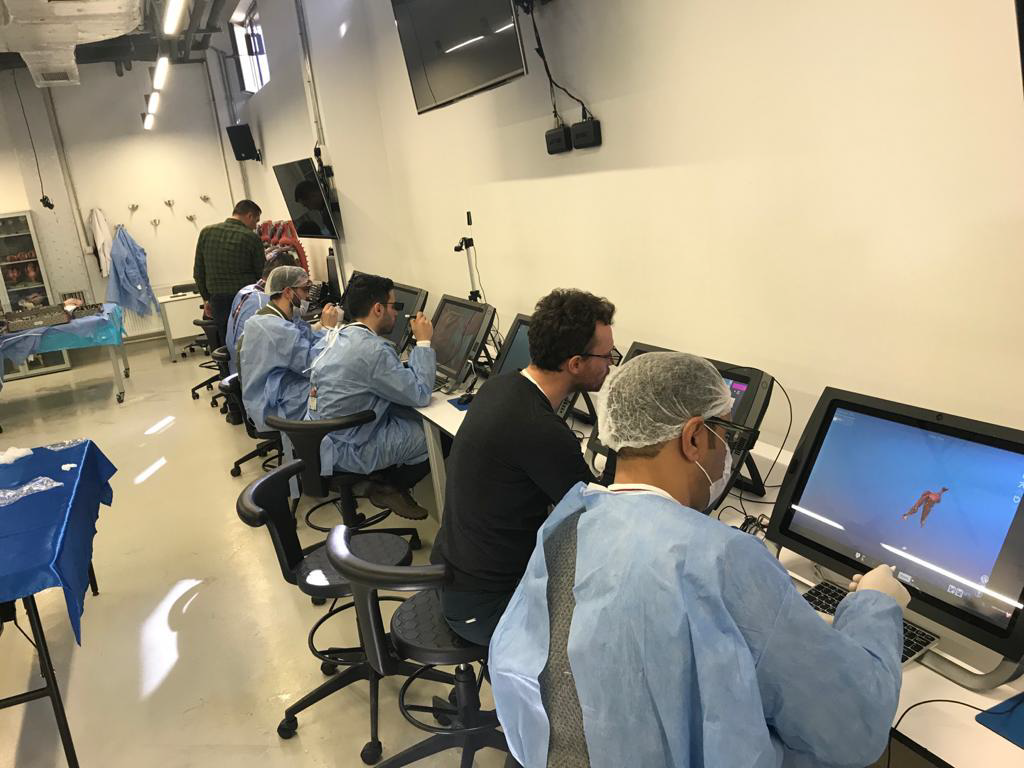
Talent test.

The camera path length, grasper path length, time of the procedure and complication as the amount of scratching of humerus, and glenoid cartilage surfaces were also recorded digitally by the simulator device which the tests were done with. The talent test can also be viewed at Figure 3.

### 2.4. Scoring system

There are three scores calculated for each participant during diagnostic and therapeutic tests: the first one is “total score”, which is composed of the overview score that is provided by the simulation software and the procedure time. A higher total score means better performance overall. Scratching of glenoid cartilage and humerus cartilage with the equipment is considered as a complication and makes up the “safety score”, higher score means lower number of complications. Finally the “economy score” reflects the economical use of the camera and grasper during the procedure. Camera path length and grasper path length are calculated by the simulator and a higher economy score means shorter path length.

### 2.5. Statistical analysis

In descriptive statistics concerning continuous data, average standard deviation, median, minimum, and maximum values have been used and percent values has been used with discrete data. Shapiro–Wilk test has been used to examine if continuous data corresponds with normal distribution.

For comparison of data, that corresponds with normal distribution, between the experiment and control groups t test has been used. For comparison of the data that does not correspond with normal distribution, Mann–Whitney U test has been used.

IBM SPSS Statistics 20 (IBM Corp., Armonk, NY, USA) program has been used for evaluations and p < 0.05 is accepted as threshold for statistical significance.

### 2.6. Ethics

Written informed consents were obtained from all participants who agreed to join the study.

Cadaver training was done at the Anatomy Laboratory of the TOBB University Faculty of Medicine with their approval. 

Written informed consents were obtained for the use of photographs.

### 2.7. Data availability

The datasets generated during and/or analysed during the current study are available from the corresponding author on reasonable request.

## 3. Results

There was not any statistically significant difference spotted (p > 0.05) between Group 1 and 2 in means of total score and simulation overview score. However, Group 2 had statistically significant higher simulation overview procedure time values than Group 1 (p < 0.05) (Table 3), the meaning of which is participants trained with the simulator completed the given tasks in a shorter period of time. 

**Table 3:** Comparison in means of general evaluation.

	Group 1 (n = 18)	Group 2 (n = 16)		
	Mean ± S.D.Median (Min–Max)	Mean ± S.D.Median (Min–Max)	t/U	p
Total score	93.56±8.6295 (81–107)	89.81±12.6791 (65v111)	1.015*	0.318
Simulation overviewscore	19.89 ± 0.3220 (19–20)	20.00 ± 0.0020 (20–20)	128.000**	0.597
Simulation overviewProcedure time	97.62 ± 35.59103.88 (33.60–152.85)	121.34 ± 12.17122.51 (78.62–129.99)	78.000**	0.022

* t test.

There was not any statistically significant difference spotted (p > 0.05) between Group 1 and 2 in means of safety scores in general. Further evaluation revealed that no statistically significant difference spotted (p > 0.05) between two groups in means of scratching of glenoid cartilage values and score, scratching of humerus cartilage score. Nevertheless, Group 2 had statistically significant higher scratching of humerus cartilage values than Group 1 (p < 0.05) (Table 4), which means that participants trained with simulation has less scratching done on the humerus cartilage than the participants trained on cadaver.

**Table 4:** Comparison in means of safety values.

	Group 1(n = 18)	Group 2(n = 16)		
	Mean ± S.D.Median (Min–Max)	Mean ± S.D.Median (Min–Max)	t/U	p
Safety score	19.93 ± 0.3820 (19–20)	19.56 ± 0.5120 (19–20)	105.000**	0.187
Scratching of glenoid cartilage	0.012 ± 0.0130.006 (0–0.044)	0.021 ± 0.0180.142 (0.001–0.069)	94.000**	0.088
Scratching of glenoid cartilage score	10.00 ± 0.0010 (10–10)	9.88 ± 0.3410 (9–10)	126.000**	0.551
Scratching of humerus cartilage	0.029 ± 0.0170.027 (0.000–0.060)	0.043 ± 0.0180.038 (0.017–0.089)	-2.095*	0.044
Scratching of humerus cartilage score	9.83 ± 0.3810 (9–10)	9.69 ± 0.4810 (9–10)	123.000**	0.484

* t test.

There was not any statistically significant difference spotted (p > 0.05) between Group 1 and 2 in means of economy scores in general, camera path length value and scores, grasper path length values and scores. (Table 5)

**Table 5:** Comparison in means of distances covered by tools values.

	Group 1(n = 18)	Group 2(n = 16)		
	Mean ± S.D.Median (Min–Max)	Mean ± S.D.Median (Min–Max)	t/U	p
Economy score	15.72 ± 4.4017 (9–20)	14.31 ± 3.5315 (7–20)	112.000**	0.281
Camera path length (cm)	82.70 ± 62.5277.42 (13.18–278.17)	97.56 ± 37.0796.33 (55.05–187.64)	106.000**	0.198
Camera path length score	8.22 ± 2.719 (0–10)	7.81 ± 2.298 (2–10)	120.500**	0.422
Grasper path length (cm)	133.97 ± 72.72115.10 (51.32–287.10)	165.29 ± 71.49151.46 (83.03–354.32)	89.000**	0.059
Grasper path length score	7.50 ± 3.429 (0–10)	6.50 ± 3.017 (0–10)	99.000**	0.126

* t test.

When two groups were compared about their diagnostic metrics (detailed visualization done inside glenohumeral and subacromial) there was not any statistically significant difference spotted (p > 0.05) between two groups in means of glenohumeral and subacromial detailed visualization scores. Further evaluation brings no statistically significant difference was spotted (p > 0.05) between groups in means of visualization values and scores of biceps tendon, supraspinatus, infraspinatus, subscapularis, humerus, glenoid cartilage, dorsal labrum, superior labrum, anterior medial labrum, inferior labrum, acromion, coracoacromial ligament, acromioclavicular joint (Table 6, Table 7). 

**Table 6:** Comparison in means of detailed visualization (diagnostic metrics).

	Group 1(n = 18)	Group 2(n = 16)		
	Mean±S.D.Median (Min–Max)	Mean±S.D.Median (Min–Max)	t/U	p
Detailed visualizationscore	37.33 ± 9.0137.50 (23–50)	33.50 ± 8.9934.50 (11–48)	1.240*	0.224
Biceps tendon(%)	0.962 ± 0.0881 (0.652–1.00)	0.920 ± 0.2290.995 (0.075–1.00)	114.000**	0.313
Biceps tendon score	4.94 ± 0.245 (4–5)	4.69 ± 1.255 (0–5)	142.500**	0.959
Supraspinatus(%)	0.359 ± 0.2730.350 (0.00–0.728)	0.306 ± 0.2510.260 (0.00–0.750)	126.500**	0.551
Supraspinatus score	2.11 ± 2.370.5 (0–5)	1.56 ± 2.190 (0–5)	130.000**	0.646
Infraspinatus(%)	0.188 ± 0.2380.112 (0.00–0.781)	0.099 ± 0.1470.033 (0.00–0.437)	120.000**	0.422
Infraspinatus score	1.28 ± 1.870 (0–5)	0.63 ± 1.410 (0-4)	108.500**	0.224
Subscapularis(%)	0.682 ± 0.1740.724 (0.209–0.868)	0.719 ± 0.1540.741 (0.440-0.948)	129.000**	0.621
Subscapularis score	4.50 ± 1.465 (0–5)	4.88 ± 0.505 (3-5)	136.000**	0.798
Humerus(%)	0.480 ± 0.2030.517 (0.119–0.869)	0.380 ± 0.1820.347 (0.173-0.889)	103.000**	0.164
Humerus score	4.06 ± 1.555 (0–5)	3.37 ± 1.594 (1-5)	99.500**	0.126
Glenoid cartilage(%)	0.839 ± 0.2480.918 (0.028–1.00)	0.801 ± 0.2520.924 (0.294-1.00)	134.000**	0.746
Glenoid cartilage score	4.39 ± 1.465 (0–5)	3.81 ± 1.905 (0-5)	120.500**	0.422
Dorsal labrum(%)	0.595 ± 0.3920.751 (0.00–1.00)	0.492 ± 0.4080.469 (0.00-0.997)	121.000**	0.443
Dorsal labrum score	3.06 ± 2.515 (0–5)	2.44 ± 2.532 (0-5)	122.500**	0.463
Superior labrum(%)	0.937 ± 0.1461 (0.451–1.00)	0.873 ± 0.3141 (0.022-1.00)	136.000**	0.798
Superior labrum score	4.83 ± 0.715 (2–5)	4.38 ± 1.715 (0-5)	133.000**	0.721
Anterior medial labrum(%)	0.947 ± 0.2241 (0.487–1.00)	0.998 ± 0.0061 (0.976-1.00)	143.500**	0.986
Anterior medial labrum score	4.72 ± 1.185 (0–5)	5.00 ± 0.005 (5-5)	136.000**	0.798
Inferior labrum(%)	0.585 ± 0.3390.655 (0.00–971)	0.522 ± 0.2790.594 (0.00-868)	120.500**	0.422
Inferior labrum score	3.44 ± 2.205 (0±5)	2.75 ± 2.444 (0±5)	122.500**	0.463

**Table 7:** Comparison in means of detailed visualization (diagnostic metrics).

	Group 1(n = 18)	Group 2(n = 16)		
	Mean ± S.D.Median (Min–Max)	Mean ± S.D.Median (Min–Max)	t/U	p*
Subacromial Detailed visualization score	0.78 ± 3.300 (0–14)	2.44 ± 5.140 (0–15)	116.000**	0.347
Acromion(%)	0.084 ± 0.2370 (0.00–1.0)	0.169 ± 0.3090.002 (0.00–0.986)	110.000**	0.251
Acromion score	0.28 ± 1.180 (0–5)	0.88 ± 1.890 (0v5)	125.500**	0.528
Coracoacromial ligament(%)	0.050 ± 0.1430.001 (0.00–0.542	0.150 ± 0.2920.027 (0.00-0.961)	92.000**	0.075
Coracoacromial ligament score	0.22 ± 0.940 (0–4)	0.63 ± 1.710 (0–5)	133.000**	0.721
Acromioclavicularjoint(%)	0.060 ± 0.1410 (0.00–0.560)	0.140 ± 0.2650 (0.00-0.729)	131.000**	0.670
Acromioclavicular joint score	0.28 ± 1.180 (0–5)	0.94 ± 2.020 (0–5)	125.000**	0.528

* t Test

## 4. Discussion

According to the data gathered and analyzed from our study, it is safe to say that training done with the cadavers is not superior to training done with the VR simulation in means of talent improvement. Moreover, participants completed the given tasks in a shorter time period together with less complication when trained with VR simulation. Together with the other advantages of virtual reality, it might replace the cadavers for training purposes as they have shown their importance even more with the Covid-19 Pandemic.

**Figure 4:**
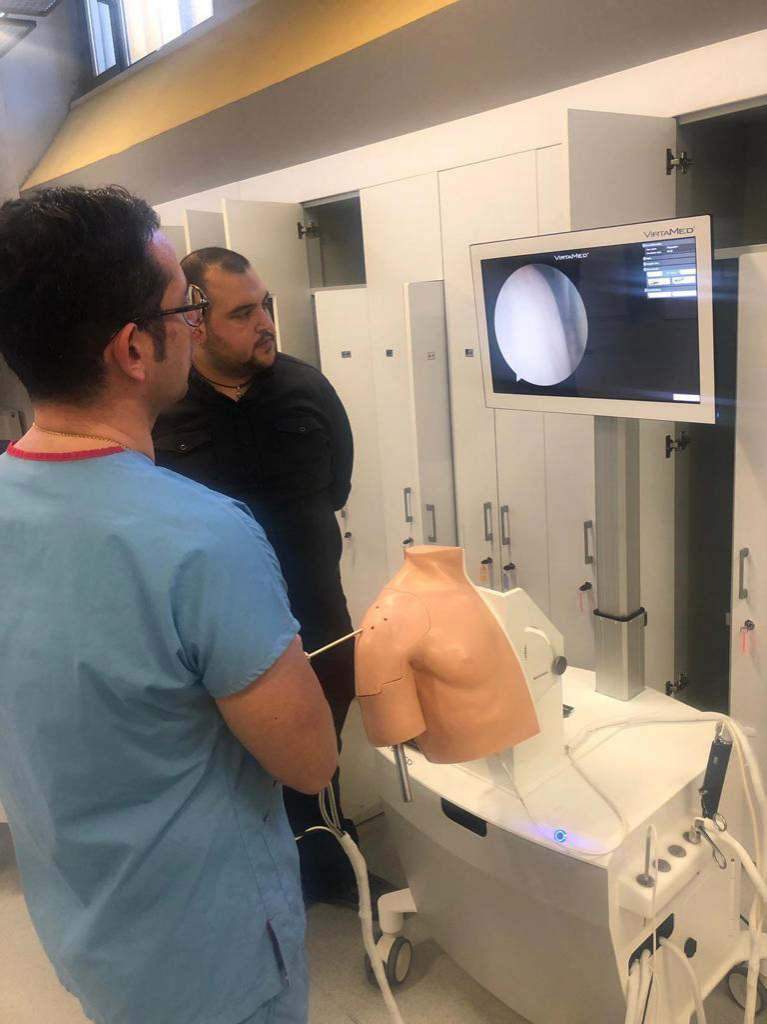
Theoretical and practical educational methods and alternatives for them during and after the pandemic

It is obvious that precautions taken for the pandemic would last for a long time [14]. Which highlights the importance of less crowded, individualized educational models in medical education just as every other field. Distance learning is promoted for theoretical education in accordance with this goal. For the fields where practical training and hands-on education is an essential part, virtual reality simulators can be considered as the best solution (Figure 4).

VR simulators are holding the upper hand in many ways. They offer the freedom of making mistakes in a risk-free environment, and it is a well-known fact that rehearsal is a key factor for learning. Trainees would learn the required basic motor skills before entering the operating room by getting familiar with equipment and tasks, which would enable them to focus on learning more complex skills during surgeries [15]. Residents with simulator training reach a minimum proficiency level in a shorter time when compared with students without simulation training [16]. Another study highlights that when residents used VR for training, they had higher levels of self-confidence [17] with crediting the holistic experience of VR training. It has also been agreed by the majority of the participants during this study and another study [18] that VR simulation trainings should take place as most valuable during the first year of residency curriculum which is also stated that, residents trained with simulators show greater skills compared with the residents not trained with simulator [19]. With technology improving, new VR simulators are being made which has construct, face, and content validity [20], which offers a chance to enhance the training even further. Using VR simulators requires less time to achieve the state of full competency when compared with real-life training; 1 h of training done with VR Simulators saves approximately 30 min to the trainee as the study shows [21].

When Covid-19 pandemic comes under control and the world returns to normal life, there will be setbacks in many fields, and medicine will be one of them. It is highly possible that hands-on practice might be disrupted long after this pandemic ends. This would have detrimental effects on specialties that require practical experience, especially for future surgeons. Authorities have seen that the educational system is vulnerable to such effects. Therefore, there will be a transition from training done with a crowded population in big lecture halls into training models done with a small audience. This would further increase the importance of Virtual Reality Simulators as they offer repeated training opportunities without any supervisor together with the opportunity of standardization around the globe. In order to prepare our medical and surgical education system for future possible pandemics, virtual reality simulators are offering an almost perfect solution.

Virtual reality simulators have some disadvantages, and there is always room for further improvements. For instance, the initial set-up cost for one device is quite high. It is not possible to mimic the pathologies with one hundred percent accuracy. There are minor anatomic variations and the trainee cannot experience them on VR simulation. Due to limitations of our study, we conducted training and tests on the same VR simulator device. Similar study to be designed with distinct simulators would provide more valuable data. We also recommend further studies with a bigger cohort.

## 5. Conclusion

To the best of our knowledge, this study is the first one to compare VR simulators with cadavers for surgical education in an objective manner, while using qualitative and quantitative data. According to this study it is possible to state that VR simulators are just as effective as cadavers in means of a training subject. As medical education will face a total change all around the world after COVID-19 pandemic, this study has the potential to be an important guide during and after this period.

## 6. Declarations

### 6.1. Author contributions

All authors have had equal contributions during every stage of the study.

### 6.2. Financial disclosure summary

No benefits in any form have been received or will be received from a commercial party related directly or indirectly to the subject of this article.

### 6.3. Consent to participate and for publication

All participants who agreed to join the study have signed the informed consents.

## Informed consent

Written informed consents were obtained from all participants who agreed to join to 10 the study. Written informed consents were also obtained for the usage of photographs.

## References

[ref1] (2020). Novel human coronavirus (SARS-CoV-2): A lesson from animal coronaviruses. Veterinary Microbiology.

[ref2] (2020). Aerosol and surface stability of SARS-CoV-2 as compared with SARS-CoV-1.

[ref3] (2020). SARS-CoV-2 and viral sepsis: observations and hypotheses. Lancet.

[ref4] (2011). When simulation in surgical training meets virtual reality. Hellenic Journal of Surgery.

[ref5] (1998). The importance of surgeon experience for clinical and economic outcomes from thyroidectomy. Annals of Surgery.

[ref6] (2004). The related outcome and complication rate in primary lumbar microscopic disc surgery depending on the surgeon’s experience: comparative studies. The Spine Journal.

[ref7] (2010). Virtual reality in orthopaedics: is it a reality?. Clinical Orthopaedics and Related Research.

[ref8] (2007). Coordinated multiple cadaver use for minimally invasive surgical training. Journal of the Society of Laparoscopic & Robotic Surgeons.

[ref9] (2014). An Economical Approach to Teaching Cadaver Anatomy: A 10-Year Retrospective. The American Biology Teacher.

[ref10] (2002). Infective agents in fixed human cadavers: a brief review and suggested guidelines. The Anatomical Record.

[ref11] (2015). Cadaveric Training: Fresh Frozen and Thiel Embalmed.

[ref12] (2018). Efficacy of standardized training on a virtual reality simulator to advance knee and shoulder arthroscopic motor skills. BMC Musculoskeletal Disorders.

[ref13] (1982). Length of operation and morbidity: is there a relationships? Plastic.

[ref14] (2020). the transmission dynamics of SARS-CoV-2 through the postpandemic period. Science.

[ref15] (2005). Virtual reality simulation for the operating room: proficiency-based training as a paradigm shift in surgical skills training. Annals of Surgery.

[ref16] (2018). Efficacy of a Virtual Arthroscopic Simulator for Orthopaedic Surgery Residents by Year in Training. Orthopaedic Journal of Sports Medicine.

[ref17] (2018). Effectiveness of Immersive Virtual Reality in Surgical Training-A Randomized Control Trial. Journal of Oral and Maxillofacial Surgery.

[ref18] (2015). Validation of the ArthroS virtual reality simulator for arthroscopic skills. Knee Surgery.

[ref19] (2014). The internal validity of arthroscopic simulators and their effectiveness in arthroscopic education. Knee Surgery.

[ref20] (2019). Validation of the Hip Arthroscopy Module of the VirtaMed Virtual Reality Arthroscopy Trainer.

[ref21] (2018). Does virtual reality simulation have a role in training trauma and orthopaedic surgeons?. The Bone & Joint Journal.

